# Book Reviews

**Published:** 1873-11-01

**Authors:** 


### Book Reviews.

Lectures on Clinical Medicine. By A. Trousseau, late Professor of Clinical Medicine in the Faculty of Medicine, Paris; Physician to the Hotel Dieu, etc., etc., etc., etc. Translated from the third revised and enlarged edition, by Sir John Rose Cormack, M.D., F.R.S.E., Fellow of the Royal College of Physicians of Edinburgh, etc., etc., etc.; and P. Victor Bazine, M.D., Assistant Physician to the National Hospital for the Paralyzed, etc., etc. Complete in two volumes. Philadelphia: Lindsay & Blakiston, 1873. For sale by Jansen, McClurg & Co., Chicago.

Here we have two volumes of nearly 1,000 pages each, embracing the views of one of the most celebrated clinical teachers of the present time. The American publishers have given us a reprint of the English edition, distributing the lectures translated by Dr. Bazine, in their proper places in the order of their delivery, and omitting his notes. The lectures of A. Trousseau, both in the French and in the English translations, have been sufficiently long before the profession to be well known and highly appreciated. In the French edition, of which the present is a translation, some of the lectures had been re-written; others had been revised, and much new matter added. The present volumes may be regarded, therefore, as presenting the completion of the work of the late illustrious teacher. They contain a great fund of knowledge on almost anything pertaining to diseases and their treatment, presented in a clear and pleasing style. We fully commend the work to our readers as worthy of their careful study.

Skin Diseases, their Description, Diagnosis and Treatment. By Tilbury Fox, M.D. Second American, from third London edition. New York: Wm. Wood & Co. For sale by Jansen, McClurg & Co., Chicago.

This, the leading standard work on Skin Diseases, both in England and America, requires no extended notice, its merits being already familiar to the profession.

In this new edition the matter has been considerably altered, re-arranged, and such additions made as to include the latest ideas and advancements. Sixty-seven new illustrations have been added; also a cutaneous pharmacopœia and a glossarial index.

The following works, received through Messrs. Jansen, McClurg & Co., will be noticed in our next:

Chemistry, Inorganic and Organic, with experiments. By Charles Loudon Bloxom. Philadelphia: H. C. Lea.

A Manual of Medical Jurisprudence. By Alfred Swain Taylor, M.D., F.R.S. Edited by John J. Reese, M.D. Philadelphia: H. C. Lea.

A Practical Treatise on the Diseases of the Ear, including the Anatomy of the Organ. By D. B. St. John Roosa, M.A., M.D. New York: Wm. Wood & Co.,

Handbook of Physiology. By Wm. S. Kirkes, M.D. Edited by Remount Baker, F.R., C.S. With 248 illustrations. A new American, from the eighth enlarged English edition. Philadelphia: H. C. Lea, 1873.

